# *In silico* evaluation of the mechanical stimulation effect on the regenerative rehabilitation for the articular cartilage local defects

**DOI:** 10.3389/fmed.2023.1134786

**Published:** 2023-03-07

**Authors:** Valentin L. Popov, Aleksandr M. Poliakov, Vladimir I. Pakhaliuk

**Affiliations:** ^1^Institute of Mechanics, Technische Universität Berlin, Berlin, Germany; ^2^Polytechnic Institute, Sevastopol State University, Sevastopol, Russia

**Keywords:** articular cartilage, osteoarthritis, articular stem cell implantation, autologous chondrocyte implantation, mechanical tissue stimulation, articular cartilage regenerative rehabilitation

## Abstract

Osteoarthritis is one of the most severe diseases of the human musculoskeletal system, and therefore, for many years, special attention has been paid to the search for effective methods of its treatment. However, even the most modern methods only in a limited number of cases in the early or intermediate stages of osteoarthritis lead to positive treatment results. In the later stages of development, osteoarthritis is practically incurable and most often ends with disability or the need for joint replacement for a large number of people. One of the main reasons hindering the development of osteoarthritis treatment methods is the peculiarities of articular cartilage, in which there is practically no vascular network and tissue homeostasis is carried out mainly due to the diffusion of nutrients present in the synovial fluid. In modern medicine, for the treatment of osteoarthritis, tissue engineering strategies have been developed based on the implantation of scaffolds populated with chondrogenic cells into the area of the defect. *In vitro* studies have established that these cells are highly mechanosensitive and, under the influence of mechanical stimuli of a certain type and intensity, their ability to proliferate and chondrogenesis increases. This property can be used to improve the efficiency of regenerative rehabilitation technologies based on the synergistic combination of cellular technologies, tissue engineering strategies, and mechanical tissue stimulation. In this work, using a regenerative rehabilitation mathematical model of local articular cartilage defects, numerical experiments were performed, the results of which indicate that the micro-and macro environment of the restored tissue, which changes during mechanical stimulation, has a significant effect on the formation of the extracellular matrix, and, consequently, cartilage tissue generally. The results obtained can be used to plan strategies for mechanical stimulation, based on the analysis of the results of cell proliferation experimental assessment after each stimulation procedure *in vivo*.

## 1. Introduction

In the process of life in its natural environment, a person is constantly under the influence of external forces and adapts to them. When the environment changes as a result of homeostasis, his body adapts to the changed conditions, which is accompanied by a change in the properties of tissues and organs. Thus, for example, under conditions of weightlessness, a local loss of bone mass occurs due to the activation of resorption as a result of reactions to the disappearance of mechanical stress and rearrangement in the hierarchy of ion and volume regulation (1). Therefore, it is quite reasonable to assume that these changes are predetermined by the evolution of the musculoskeletal system of terrestrial vertebrates in the earthly gravity field and are determined biomechanically. But, as is known, a decrease in the load on the bone is accompanied not only by a decrease in bone mass, but also by a change in the relationship of the entire cellular aggregate and the extracellular matrix (ECM) of the tissue (2). Similar processes occur not only in bone, but also in other tissues, and not only when the force of gravity changes. It has been established that various kinds of physical influences affect the physiological and reparative regeneration of tissue defects resulting from injuries or diseases. Moreover, the course of these processes depends not only on the nature of the external influence, but also on many other factors, including the physical condition of the patient, his gender, age and even race, as well as the type and size of the tissue defect, the strategy of pharmacotherapy, etc. In this regard, it is quite natural for specialists in the area of regenerative medicine to understand how and why rehabilitation medicine, which uses physical influences in its practice, can help restore damaged tissues of a particular patient. In recent years, this desire has led to the creation of a new direction in medical science-regenerative rehabilitation, the essence of which is to find and practically implement the best conditions for the restoration of damaged tissues through the parallel use of advanced methods of regenerative and rehabilitation medicine. However, despite a number of optimistic results obtained using regenerative and rehabilitation approaches, there are still many questions and problems that need to be addressed for the development of this area of science and the creation on its basis of effective technologies for the treatment of diseases associated with various types of damage to tissues and organs.

One of the main problems hindering the introduction of regenerative rehabilitation technologies into medical practice is the lack of a complete understanding of the cells and tissues response to physiological effects and the lack of theoretical and experimental data for their systematization. Such technologies should take into account not only the type and biophysical state of the restored tissue, but also the features of its interaction with surrounding tissues, which requires considering many factors that affect the intensity and quality of restoration. In this regard, of particular relevance is the mathematical simulation of the regenerative rehabilitation processes, which is necessary to assess the significance of the parameters that determine their course and use the results obtained in planning experimental studies *in vivo*.

In this paper, we study a mathematical model of regenerative rehabilitation the local articular cartilage defects, taking into account the features of this type of skeletal connective tissue, which are well studied and described in detail in many literature sources (3–7). It also takes into account the responses of chondrocytes and progenitor chondrocytes observed in experiments *in vitro* to a wide range of mechanical stimuli, including tensile, compressive, shear deformations, fluid flow, hydrostatic and osmotic pressure (8–10). Mesenchymal stem cells (MSCs), capable of chondrogenic differentiation, also respond to these stimuli, and therefore represent a potential source of chondroblasts, from which, in turn, chondrocytes are formed, the main function of which in cartilage tissue homeostasis is the synthesis and release of intercellular substance components consisting of water, proteoglycan aggregates, glycoproteins and minerals (11–13). As a result of this activity, chondrocytes wall themselves up in specific areas of the ECM–lacunae, thereby providing interstitial cartilage growth and its potential ability to regenerate.

It is known that the responses of cells to mechanical influences are different and cause many changes and sensations, the study of which has received much attention for a long period of time (14–19). However, it is still not fully understood how exactly mechanical signals are transmitted to individual cells, how versatile the mechanisms of mechanotransduction are, and whether there is redundancy between possible signal transduction pathways. The mathematical model studied in this work takes into account the experimentally observed effect of chondrogenic cells physical stimulation on their proliferation, differentiation, viability, and ECM formation, both with and without regard to the biochemical processes that determine these phenomena. At the same time, the model itself is built considering the following conditions (20, 21):

- healthy cartilage in the process of life is subjected to a complex load that ensures its stress–strain state;
- an important factor determining the viability and regeneration of articular cartilage under *in vivo* conditions is its dynamic loading;
- when an external load is applied to the cartilage, due to mechanotransduction, biochemical signals are activated in it that regulate both anabolic and catabolic processes, including the synthesis of matrix proteins, transcription factors, growth factors, proteases and protease inhibitors;
- the balance between these processes is largely achieved due to the external load perceived by the joint and depends on its type and intensity.
- The model also does not contradict the assessments currently accepted in the scientific community of the influence of various factors on homeostasis and the function of articular cartilage (22):
- excessive mechanical stress on the articular cartilage leads to mitochondrial dysfunction, hypertrophy of chondrocytes, degradation of collagen, a decrease in the level of adenosine triphosphate and the formation of reactive oxygen species;
- proper mechanical stimulation of MSCs increases viability and enhances chondrogenesis of cells, promotes collagen synthesis, increased ECM formation and organization of a network of fibers in tissue-engineered cartilage structures;
- growth factors (BMP, TGF, IGF, etc.) maintain the integrity of the articular cartilage, promote the secretion of glycosaminoglycans, the expression of chondrogenic genes, the proliferation and differentiation of MSCs into chondrocytes;
- pro-inflammatory cytokines (Il-1β, TNF-α) inhibit the expression of genes responsible for the formation of cartilage ECM and chondrocyte phenotype, as well as the differentiation of MSCs into chondrocytes.

It is clear that it is practically impossible to simultaneously take into account in the mathematical model all the many factors listed above, considering among other things, their possible mutual influence. In addition, even considering these factors conditionally independent, it is very difficult to formulate their mathematical representations and determine the corresponding numerical values. For example, it is possible to experimentally determine quantitative indicators of the increase in viability, cell proliferation, the rate of chondrogenesis, collagen synthesis and ECM depending on the location, type and intensity of mechanical stimulation of MSCs, the mathematical representation of which, obviously, should be based on a synergistic combination of heterogeneous natural phenomena (mechanical, chemical, biological) at different levels of detail. These phenomena are of a random nature, and their occurrence essentially depends on the state of the medium. Therefore, taking into account the understanding of their essence, the mathematical model of tissue regenerative rehabilitation can be reasonably simplified by using the average values of the parameters corresponding to the experimental data. At the same time, the nature of the occurrence of phenomena and the mechanisms that determine the change in the parameters of the medium state remain important, but are not considered directly in the process of their determination.

The main goal of this work is evaluating the effect of mechanical stimulation on the effectiveness of regenerative rehabilitation for local articular cartilage defects using various cell technologies and tissue engineering strategies as a result of studying a mathematical model with parameters determined as a result of experimental studies available in the literature.

## 2. Materials and methods

### 2.1. Basic strategies for articular cartilage defect repair

Articular cartilage covers the surfaces of bones in diarthrotic joints, ensuring their relative movement with low energy consumption for friction and acting as a shock absorber for external loads. At the same time, it is able to deform with an increase in the area of the contact surface, which helps to reduce pressure on the bones that form the joint. Articular cartilage has a two-phase structure and possesses viscoelastic properties that provide stress relaxation during compression and resistance to damage from external loads (4).

The thickness of the cartilage on the surfaces of the bones that form human joints ranges from 1 mm to 4 mm and its properties vary depending on the depth, forming four pronounced zones: superficial, intermediate (middle), deep and calcified, in which the shape of chondrocytes changes from flat to spherical (6). Collagen fibers in the superficial zone are parallel to the articular surface, in the intermediate zone they are randomly oriented in different directions, and in the deep zone they are organized perpendicular to the articular surface so that they penetrate into the calcified zone, thereby ensuring the structural stability of the articular cartilage on the subchondral bone.

Articular cartilage degradation can occur because of injury, disease, or constant mechanical stress and is classified into three main types: superficial destruction (damage to the ECM), partial thickness defects (does not extend into subchondral bone), and full-thickness defects (penetrate deep into subchondral bone) (23). Only with superficial destruction of the articular cartilage, viable chondrocytes can form clusters and are potentially capable of independently synthesizing a new matrix. With deep defects of all types, cartilage self-healing is practically excluded, therefore, for the purpose of their therapeutic or surgical restoration, a number of strategies have been developed, which, however, in most cases also do not guarantee positive results (22, 24, 25).

Modern strategies focused on the restoration or regeneration of articular cartilage, including in osteoarthritis, involve the implantation of chondrocytes or MSCs, biodegradable scaffolds, and signaling molecules (cytokines and growth factors) into the defect area. Scaffolds in this triad are used to potentially provide biological signals that regulate cell behavior or as scaffolds in which cells must synthesize ECM, and signaling molecules to stimulate recruitment, differentiation of progenitor cells, and also to direct the synthesis of the desired tissue phenotype (26).

#### 2.1.1. Autologous chondrocyte implantation technology and matrix-induced autologous chondrocyte implantation technology

Autologous Chondrocyte Implantation Technology (ACIT) is used to treat certain symptomatic articular cartilage defects in synovial joints, usually implemented in three stages (27–29). At the first of these, arthroscopy of the patient’s joint is performed with a sampling of 200–300 milligrams of cartilage, usually from the least loaded area. ECM is enzymatically removed from the harvested tissue and chondrocytes are isolated. At the second stage, these cells are grown in a specialized bioreactor under *in vitro* conditions until their number is sufficient for implantation into the defect area, which takes approximately 1.0–1.5 months. In recent years, ACIT has been improved and at this stage, modern biodegradable scaffolds or hydrogels have been used to promote the formation of a three-dimensional tissue ECM. This ACIT modification, called Matrix-Induced Autologous Chondrocyte Implantation Technology (MACIT), is becoming increasingly popular due to its cost-effectiveness compared to first-generation ACIT, as well as better cell maturation *in vitro* and better cell growth *in vivo* (30). And, finally, at the third stage, the cells grown in the bioreactor are implanted into the defect area, adapt to the new environment and form a new cartilage.

#### 2.1.2. Articular stem cell implantation technology

Relatively recently, it was found that cartilage can be restored if there are sufficient resources of MSCs in the area of the defect (31, 32). Undifferentiated MSCs, like other cells, are mechanosensitive; therefore, not only biochemical but also biomechanical factors play an important role in their chondrogenic differentiation. This is an important feature of MSCs, which are able to differentiate into different cell types during the process of committing to chondrocytes. Since the committing mechanism is a persistent repression of some and de-repression of other genes, the spectrum of functionally active genes gradually changes in cells as they develop, which determines an increasingly specific direction for their future fate. At a certain stage, the commitment leads to the fact that cells become determined with genetic programming for only one developmental path. That is, under certain conditions, these cells have the ability to differentiate along different mesenchymal lines, including cartilage (33–35). At the same time, as noted above, proteins such as fibroblast growth factors (FGFs), bone morphogenetic proteins (BMPs), etc., are involved in the regulation of MSCs chondrogenesis (36, 37). In healthy cartilage, the metabolism and renewal of chondrocytes are primarily provided by the growth factors FGF-1 and BMP-2 (38–40).

MSCs used to repair cartilage defects are obtained from various autologous tissues, including bone marrow, adipose tissue, and peripheral blood (41) and, depending on the specific pathology, are either surgically implanted into the defect or injected into the joint. This process is called Articular Stem Cell Implantation Technology (ASIT).

**Figure 1:**
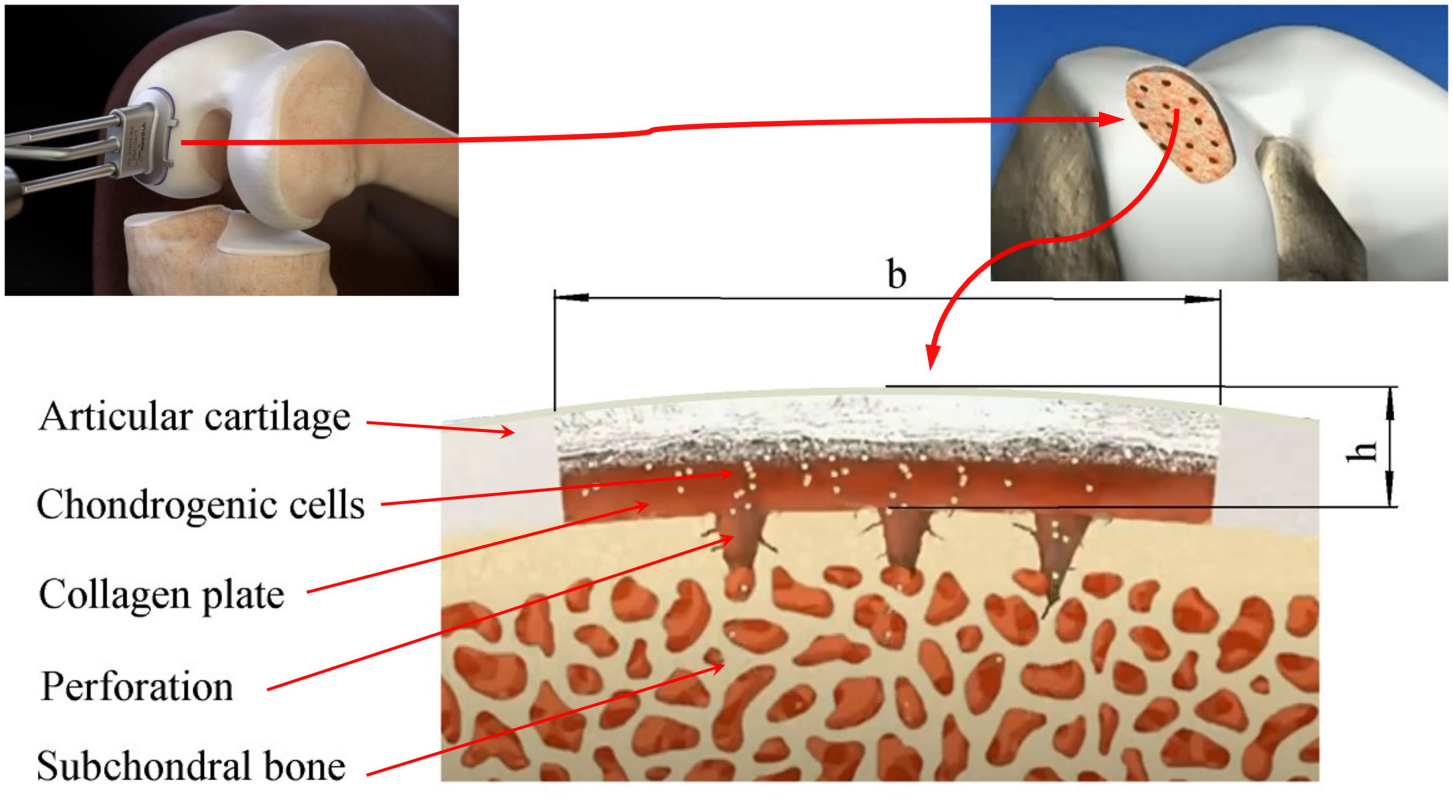
Scheme of the damaged area of the articular cartilage filled with a tissue-engineered structure based on a collagen plate placed on the subchondral bone: *b*, *h*-the maximum size and depth of the defect, respectively.

The area of a local articular cartilage defect replaced by a tissue-engineered construct supported by a collagen plate placed on the subchondral bone and the surgical procedures required for ACIT/ASIT are schematically shown in Figure 1.

### 2.2. Mechanical stimulation of chondrogenesis

In the process of life, the joints of the human lower limb during normal locomotion are subjected to cyclic compression *in vivo* with a frequency of about 1 Hz. In this case, chondrocytes are cyclically loaded with uniform pressure ranging from 3 MPa to 10 MPa (9). It is also known that certain movement and load patterns are required for the normal development of joints and articular cartilage *in vivo* (42). In this regard, the opinion has been formulated in the scientific community that mechanically generated signals play a critical role in the proliferation, differentiation, and maturation of progenitor chondrocytes and MSCs to the chondrogenic phenotype.

It has been established that compressive loading causing compression deformation of scaffolds seeded with MSCs induces a prochondrogenic and biosynthetic response, useful in the creation of implants for cartilage regeneration and repair using ASIT (14). And in more advanced bioreactors, which allow, in addition to compression, to realize shear and other components of the load, the chondrogenic response of MSCs to mechanical load not only increases, but also better mimics the *in vivo* environment, which contributes to better differentiation of chondrocytes, leads to an increase in the formation, composition and location of the ECM during cartilage regeneration (43).

But not only has a certain one-dimensional or complex stimulation contributed to the emergence of a prochondrogenic reaction of cartilage tissue. It was shown in (44) that cartilage formation *in vitro* increases under the influence of any “correct” physical stimuli that promote proliferation, differentiation of chondrogenic cells, and ECM production. For example, signals generated by oscillatory fluid flow (OFF) regulate the expression of transcription factors involved in multiple differentiation pathways (45) and promote an increase in the proliferation rate of MSCs (46). In this case, the RhoA and ROCKII proteins are activated, which ultimately also regulates the differentiation of MSCs (47). The authors of (48) demonstrated that chondrogenesis of bone marrow-derived MSCs under the action of cyclic compressive load is induced similarly to that under treatment with growth factors, and both stimuli use similar pathways for this (49). But when MSCs are subjected to the combined action of cyclic contraction and treatment with growth factors, chondrogenesis is a much more complex process. With the simultaneous action of these factors, the expression levels of aggrecan decrease compared to the action of only the last of them (50); cartilage ECM synthesis in agarose hydrogels is reduced when mechanical stimulation is initiated at the onset of growth factor-induced chondrogenesis (50, 51); cyclic compression enhances the accumulation of proteoglycans and collagen for MSCs seeded in a gelatin scaffold (52), etc. Such contradictions indicate that the nature of their interaction with the surrounding ECM is of decisive importance on the response of MSCs to the load. This, in particular, explains the experimentally observed effect that the response of MSCs to dynamic compression in the presence of growth factors depends on when the load is initiated. Mouw et al. showed that early mechanical stimulation (on day 8) reduces the expression of the aggrecan gene, and at a later date (on day 16) increases the expression of the chondrogenic gene (53). That is, the results of *in vitro* experiments indicate that the mechanoreactivity of MSCs varies depending on the stage of chondrogenesis and the development of the ECM. In addition, it was found that the maintenance of the chondrogenic phenotype of MSCs by cyclic compression depends on the concentration of growth factors, but does not disappear after the exclusion of this type of stimulation (54, 55).

Chondrogenesis in scaffolds seeded with MSCs also increases under cyclic hydrostatic pressure, as evidenced by the observed *in vitro* increase in the content of proteoglycan and collagen in the chondrogenic culture medium (56). In (57), the authors note that this type of mechanical stimulation also enhances Sox9 mRNA expression, as well as type II collagen and aggrecan mRNA expression in MSCs aggregates maintained in chondrogenic conditions compared to unloaded cells (57). In addition, they found that different values of hydrostatic pressure (0.1 MPa, 1.0 MPa, 10.0 MPa) had different effects on the regulation of chondrogenesis of MSC aggregates, with greater expression of collagen type II mRNA and accumulation of collagen observed at 10 MPa (58). At the same time, it was demonstrated in (59) that hydrostatic pressure has practically no effect on the expression of chondrogenic genes or the accumulation of ECM in MSC aggregates, both in the presence and in the absence of growth factors FGF-1 or BMP-2.

The above information about the reactions of cartilage tissue to mechanical stimulation under various conditions is not systematic and does not give a complete picture of the transformations occurring in the tissue. However, it indicates that biological processes in a tissue are determined not only by its biochemical environment, but also by its biomechanical one. At the same time, the regulation of the biomechanical environment has a significant effect on the course of both anabolic and catabolic processes. In this regard, it is quite reasonable to assume that there is such a state of damaged tissue, induced biomechanically, in which its self-healing is possible. The most striking example here is the state of the tissue, in which its physiological regeneration occurs in the natural habitat of a healthy biological object. Therefore, one of the main tasks of regenerative rehabilitation is to establish such a biomechanical environment of the damaged tissue, in which the processes of its physiological regeneration are initiated. This is a very complex problem, the solution of which depends not only on a large number of parameters, but also on the range and nature of their change when exposed to tissue stimuli of different nature and intensity. In addition, there is a high probability that mechanical and other stimuli are synergistically related to each other and their contribution to the course of tissue and cellular processes is characterized by a high degree of uncertainty, and most modern studies in the field of mechanobiology consider cell responses to each stimulus separately (60). Along with the incompletely understood molecular mechanisms that determine mechanotransduction, this leads to a difficult understanding of regenerative processes in tissues subjected to stimulation and their use in medicine. Nevertheless, even the currently known results of research in the field of mechanobiology make it possible to build and study mathematical models of various degrees of detail, representing changes in tissues and cells during various types of their stimulation.

### 2.3. Mathematical model of regenerative rehabilitation for local articular cartilage defects

A mathematical model of regenerative rehabilitation for local articular cartilage defects used by authors in this work is based on a system of differential equations of the “diffusion–reaction” type (61), similar to the model used by A. Bailón-Plaza and M.C. van der Meulen to study the healing of bone fractures (62). Sufficiently realistic results in the study of such a model were obtained by M. Lutianov and colleagues who studied the processes of cartilage tissue regeneration using cell therapy (63), as well as by K. Campbell and colleagues when studying ACIT and ASIT in the presence of growth factors FGF-1 and BMP-2 (64, 65).

ACIT and ASIT, as well as their combinations, suggest the presence of a scaffold populated by chondrocytes and/or chondrogenic cells (MSCs) in the area of the cartilage defect. If we denote the density of MSCs by $C_{S}$, then the mathematical model of its change in time, taking into account the fact that $C_{S}{\;}_{0}$ is the threshold density, can be represented by the differential equation (63):


(1) $$\begin{array}{l} \frac{\partial C_{S}}{\partial t}=\underset{diffusion}{\underset{︸}{\nabla \left[D_{S}\nabla C_{S}\right]}}+\underset{proliferation}{\underset{︸}{p_{1}C_{S}\frac{n}{n+n_{0}}H\left(n-n_{1}\right)}}\\ \;-\underset{differentiation}{\underset{︸}{p_{2}C_{S}H\left(C_{S}-C_{S}{\;}_{0}\right)}}-\underset{death}{\underset{︸}{p_{3}C_{S}H\left(n_{1}-n\right)}}, \end{array}$$

where $\nabla =\frac{\partial }{\partial x}\vec{i}+\frac{\partial }{\partial y}\vec{j}+\frac{\partial }{\partial z}\vec{k}$ is the Laplace operator; $\begin{array}{l} \nabla C_{S}=\frac{\partial C_{S}}{\partial x}\vec{i}+\frac{\partial C_{S}}{\partial y}\vec{j}+\frac{\partial C_{S}}{\partial z}\vec{k}=\vec{grad}C_{S};\nabla \vec{grad}C_{S}=div\vec{grad}C_{S}\\ \;=\frac{\partial^{2}C_{S}}{\partial x^{2}}+\frac{\partial^{2}C_{S}}{\partial y^{2}}+\frac{\partial^{2}C_{S}}{\partial z^{2}}; \end{array}$$D_{S}$ is the probability diffusion coefficient of MSCs; nis the concentration of nutrients that provide tissue homeostasis ($n_{0},n_{1}\;$are the threshold and critical concentrations, respectively); $p_{1},p_{2},p_{3}$ are the coefficients that determine the proliferation, differentiation and death of MSCs, respectively; $H\left(C_{S}-C_{S_{0}}\right)=\{\begin{array}{c} 0,C_{S}\leq C_{S_{0}}\\ 1,C_{S}>C_{S_{0}} \end{array},H\left(n-n_{1}\right)=\{\begin{array}{c} 0,n\leq n_{1}\\ 1,n>n_{1} \end{array},H\left(n_{1}-n\right)=\{\begin{array}{c} 0,n_{1}\leq n\\ 1,n_{1}>n \end{array}\;$are the Heaviside step functions.

Here and below, we use the parameters designations and state variables of the model adopted earlier by K. Campbell et al. in (64, 65).

It follows from Equation (1) that the change in $C_{S}\;$in the region of the defect is determined by four terms on the right side, which describe the processes of diffusion, proliferation, differentiation, and death of MSCs. Indeed, the increase in cell density depends on the number of MSCs implanted in the defect area using ASIT and their entry into this area as a result of diffusion from the subchondral bone. If, in this case, the concentration of nutrients in the area of the defect is greater than the critical one ($n>n_{1}$), MSCs proliferate, which also leads to an increase in their density. Otherwise, due to a lack of nutrients ($n\leq n_{1}$) a certain number of MSCs die, which leads to a decrease in their density. In addition, the decrease in $C_{S}\;$occurs due to the fact that, when the threshold density is exceeded, some MSCs differentiate into chondrocytes as a result of commitment.

Similarly, a mathematical model of changes in the density of $C_{C}\;$chondrocytes in the area of a cartilage defect can be presented (63):


(2) $$\begin{array}{l} \frac{\partial C_{C}}{\partial t}=\underset{diffusion}{\underset{︸}{\nabla \left[D_{C}\nabla C_{C}\right]}}+\underset{proliferation}{\underset{︸}{p_{4}C_{C}\frac{n}{n+n_{0}}H\left(n-n_{1}\right)}}\\ \;+\underset{differentiation}{\underset{︸}{p_{2}C_{S}H\left(C_{S}-C_{S0}\right)}}-\underset{death}{\underset{︸}{p_{5}C_{C}H\left(n_{1}-n\right)}}, \end{array}$$

where $D_{C}$ is the probability coefficient of chondrocytes diffusion; $p_{4},p_{5}\;$are the coefficients of proliferation and death of chondrocytes, respectively.

The fundamental difference between Formulas (1), (2) from each other is that the differentiation of MSCs leads to a decrease in $C_{S}$ and, simultaneously, to an increase in $C_{C}$.

Taking into account the meaning of the elements of the structure of Equations (1), (2), the mathematical model of the change in the concentration of nutrients can be represented as follows:


(3) $$\frac{\partial n}{\partial t}=\underset{diffusion}{\underset{︸}{\nabla \left[D_{n}\nabla n\right]}}-\underset{reaction}{\underset{︸}{\frac{n}{n+n_{0}}\left(p_{6}C_{S}+p_{7}C_{C}\right)}},$$

where $D_{n}$ is the probability coefficient of nutrients diffusion into the defect area from the synovial cavity; $p_{6},p_{7}\;$are the nutrient consumption constants of MSCs and chondrocytes, respectively.

That is, the nutrients that enter the defect as a result of diffusion through the surface of the articular cartilage are used to maintain the viability of MSCs and chondrocytes.

It is well known that articular cartilage is a collection of cells–chondrocytes occluded in the ECM containing collagen II, glycosaminoglycans, glycoproteins and proteoglycans (aggrecan) that bind large amounts of water. ECM elements, as well as MSCs, penetrate into the cartilage defect from the subchondral bone as a result of diffusion, which contributes to an increase in the density of the matrix *m*. In addition, the ECM density increases due to chondrocytes diffusing into the defect and differentiated from MSCs, secreting it and promoting the growth of the collagen network in the scaffold. Therefore, the mathematical model for changing the density of the matrix *m* can be represented by the equation (63):


(4) $$\frac{\partial m}{\partial t}=\underset{diffusion}{\underset{︸}{\nabla \left[D_{m}\nabla m\right]}}+\underset{reaction}{\underset{︸}{p_{8}\frac{n}{n+n_{0}}C_{C}}},$$

given that $m\leq m_{\max}$, where $D_{m}$ is the probability coefficient of ECM elements diffusion; $p_{8}$ is the ECM secretion rate; $m_{\max}$ is the maximum ECM density.

Considering the fact that chondrocytes are relatively evenly distributed in the ECM in each layer of healthy cartilage, the time to reach a certain density threshold value $m_{0}\leq m_{\max}$ can serve as a conditional criterion for the quality of the regenerative rehabilitation process for an articular cartilage defect. If it is impossible to reach the threshold value of density due to a complex of possible reasons related to the biomechanical environment of the tissue being restored, the maximum achievable ECM density in a certain period of time can be taken as a criterion for the quality of various processes.

Growth factors play an important role in maintaining the balance and regeneration of articular cartilage. But their influence on changes in the density of MSCs, chondrocytes and ECM in mechanobiological models is taken into account in an implicit form. Nevertheless, such an influence can be quite noticeable, since the change in the state variables of the regenerative rehabilitation model largely depends on the ability of chondrogenic cells to proliferate and differentiate into chondrocytes, which, as shown above, is also stimulated by growth factors. Therefore, models of changes in the concentrations of growth factors in the regenerative rehabilitation process are necessary to consider their influence on cellular processes. They can be represented in the following form (63):


(5) $$\frac{\partial g}{\partial t}=\underset{diffusion}{\underset{︸}{\nabla \left[D_{g}\nabla g\right]}}+\underset{reaction}{\underset{︸}{p_{9}C_{S}-p_{11}g}},$$

(6) $$\frac{\partial b}{\partial t}=\underset{diffusion}{\underset{︸}{\nabla \left[D_{b}\nabla b\right]}}+\underset{reaction}{\underset{︸}{p_{12}C_{C}-p_{13}b}},$$

where g,bare the concentrations of growth factors FGF-1 and BMP-2, respectively; $\left(D_{g},D_{b}\right),\left(p_{9},p_{12}\right),\left(p_{11},p_{13}\right)$ are probabilistic diffusion coefficients, production and degradation constants of growth factors FGF-1 and BMP-2, respectively.

The mathematical model represented by the system of differential equations (1–6), justified earlier and described in detail in (63), makes it possible to investigate the changes occurring in the articular cartilage defect area since the beginning of ASIT/ACIT use. However, rehabilitation procedures provided by regenerative rehabilitation protocols and including physical stimulation of the tissue are usually applied with a certain time delay in order to achieve the best results, which, as shown for example in (53), is highly desirable, because allows you to achieve the maximum effect of tissue restoration. Therefore, in the mathematical model of regenerative rehabilitation with the same delay, changes in the values of parameters determined by the nature of physical stimulation should be taken into account.

Let us assume that stimulation of a scaffold populated with chondrogenic cells according to the ASIT/ACIT protocols and placed at the site of a local cartilage defect begins at time $t=t_{1}$. Then Equations (1), (2) can be represented in the following form:


(1*) $$\begin{array}{l} \frac{\partial C_{S}}{\partial t}=\nabla \left[D_{S}\nabla C_{S}\right]+C_{S}\frac{n}{n+n_{0}}H\left(n-n_{1}\right)\left[p_{1}+\left(p_{1}^{*}-p_{1}\right)H\left(t-t_{1}\right)\right]\\ \;-C_{S}H\left(C_{S}-C_{S}{\;}_{0}\right)\left[p_{2}+\left(p_{2}^{*}-p_{1}\right)H\left(t-t_{1}\right)\right]\\ \;-C_{S}H\left(n_{1}-n\right)\left[p_{3}+\left(p_{3}^{*}-p_{3}\right)H\left(t-t_{1}\right)\right], \end{array}$$

(2*) $$\begin{array}{l} \frac{\partial C_{C}}{\partial t}=\nabla \left[D_{C}\nabla C_{C}\right]+C_{C}\frac{n}{n+n_{0}}H\left(n-n_{1}\right)\left[p_{4}+\left(p_{4}^{*}-p_{4}\right)H\left(t-t_{1}\right)\right]\\ \;+C_{S}H\left(C_{S}-C_{S}{\;}_{0}\right)\left[p_{2}+\left(p_{2}^{*}-p_{1}\right)H\left(t-t_{1}\right)\right]\\ \;-C_{C}H\left(n_{1}-n\right)\left[p_{5}+\left(p_{5}^{*}-p_{5}\right)H\left(t-t_{1}\right)\right], \end{array}$$

where $H\left(t-t_{1}\right)$ is the Heaviside step function.

Thus, the mathematical model of regenerative rehabilitation of a local articular cartilage defect is represented by a system of partial differential equations (1*, 2*, 3, 4, 5, 6).

## 3. Results and discussion

In general, the mathematical model described above can be used to study state variables that change over time in three-dimensional space. However, due to the fact that the main goal of research in this work is to study the generalized reaction of cartilage tissue in response to stimulating effects, it is sufficient to study a one-dimensional model that allows studying the change in state variables only with respect to the depth of the defect *h*. This limitation is also admissible from a geometric point of view, provided that the defect dimensions (*h* and *b*) are small and the articular surface is curvature in the area of the defect.

A number of limitations of the mathematical model are determined by the nature of the interaction between subchondral bone, chondrogenic cells, nutrients, growth factors, and ECM. In this work, it is assumed that the subchondral bone is permeable and MSCs can diffuse from it into the defect area, the flow of which is given as a function of time *f*(*t*). In practice, in order to increase the intensity of this flow, the subchondral bone is usually perforated and covered with a thin permeable collagen sheet, as shown in Figure 1. At the same time, it is assumed that the flow from the subchondral bone of chondrocytes, growth factors, nutrients and ECM elements is zero. However, if necessary, the model allows you to set them in the form of certain functions of time.

Similarly, plausible model constraints on the defect surface can be represented. It can be assumed that the fluxes of MSCs, chondrocytes, and ECM elements on the surface of the defect are equal to zero, and nutrients with a constant concentration $N_{0}\;$enter the defect from the synovial fluid. In this paper, it is assumed that the fluxes of growth factors are proportional to their concentrations with proportionality factors γ and χ, respectively.

Taking into account the above restrictions, the boundary conditions of the mathematical model have the form:

(a) on the surface of the collagen plate resting on the subchondral bone, i.e., for x=0:


(7) $$\begin{array}{l} -D_{S}\frac{\partial C_{S}}{\partial x}=f\left(t\right),D_{C}\frac{\partial C_{C}}{\partial x}=0,D_{n}\frac{\partial n}{\partial x}=0,\\ D_{m}\frac{\partial m}{\partial x}=0,D_{g}\frac{\partial g}{\partial x}=0,D_{b}\frac{\partial b}{\partial x}=0, \end{array}$$

(b) on the surface of the defect, geometrically coinciding with the surface of the articular cartilage in the area of the defect, i.e., for x=d:


(8) $$\begin{array}{l} \;D_{S}\frac{\partial C_{S}}{\partial x}=0,D_{C}\frac{\partial C_{C}}{\partial x}=0,n=N_{0},D_{m}\frac{\partial m}{\partial x}=0,\\ \;D_{g}\frac{\partial g}{\partial x}=-\gamma g,D_{b}\frac{\partial b}{\partial x}=-\chi b. \end{array}$$

The initial conditions of the mathematical model are formulated in accordance with the technology of cell therapy used in the process of regenerative rehabilitation. For example, when implementing the ASIT strategy, it is assumed that MSCs are implanted into a defect, and the cells are arranged according to a certain law $C_{S}\left(0\right)=C_{S}^{\left(0\right)}h\left(x\right)$ according to the height of the defect. If a scaffold is implanted into the defect, then the initial density of ECM is assumed to be $\left(0\right)=m_{3}+m_{s}$, where $m_{3}$ is the initial ECM density, and $m_{s}$ is the scaffold density. In this case, given the nutrient density $n\left(0\right)=N_{0}$ and zero values of other state variables at the initial time, the initial conditions are as follows:


(9) $$\begin{array}{l} C_{S}\left(0\right)=C_{S}^{\left(0\right)}h\left(x\right),C_{C}\left(0\right)=0,n\left(0\right)=N_{0},\\ \;m\left(0\right)=m_{3}+m_{s},g\left(0\right)=0,b\left(0\right)=0, \end{array}$$

where $C_{S}^{\left(0\right)}$ is the initial MSC density.

Similarly, the initial conditions for the implementation of ACIT or the combination of ASIT+ACIT can be formulated.

The equations of the mathematical model studied in this work only to a small extent characterize the relationship of state variables. To a greater extent, these connections are manifested in the disclosure of the variable parameters of the model. In this paper, the content and structure of the model parameters were adopted according to (63). So, for example, it is assumed that the probability coefficients of diffusion of MSCs and chondrocytes depend on the density of the ECM and are determined by the following expressions:


(10) $$D_{S}=D_{S0}\frac{m}{m^{2}+m_{1}^{2}},D_{C}=D_{C0}\frac{m}{m^{2}+m_{1}^{2}}$$

where $m_{1}$ is the intermediate ECM density, and $D_{S0}=2m_{1}D_{S}^{*}$ and $D_{C0}=2m_{1}D_{C}^{*}$ are the diffusion constants of MSCs and chondrocytes, calculated taking into account the maximum possible diffusion coefficients $D_{S}^{*}$ and $D_{С}^{*}$. That is, at *m* = 0, the diffusion coefficients *D_S_* and *D_C_* also tend to zero, and their maximum values $D_{S}^{*}$ and $D_{С}^{*}$ are reached at $m=m_{1}$.

The proliferation coefficients of MSCs $p_{1}$ and chondrocytes $p_{4}$ also depend on state variables and are presented as


(11) $$p_{1}=p_{1_{0}}\frac{m}{m^{2}+m_{2}^{2}}\left[1-\frac{C_{S}}{C_{Smax}}\right],$$

(12) $$p_{4}=\left(p_{4_{0}}\frac{m}{m^{2}+m_{2}^{2}}+p_{4_{00}}\frac{g}{g+g_{0}}\right)\left[1-\frac{C_{C}}{C_{Cmax}}\right],$$

where $p_{1_{0}}$ and $p_{4_{0}}$ are the proliferation constants of MSCs and chondrocytes; $p_{4_{00}}$ are the degree of chondrocytes proliferation due to growth factor FGF-1; $g_{0}$ is the reference concentration of FGF-1. At the same time, the rate of synthesis of ECM $p_{8}$ decreases as its density increases and can be represented by a linear dependence (62).


(13) $$p_{8}=p_{8_{0}}-p_{8_{1}}m,$$

where $p_{8_{0}}$ is the ECM expression constant; $p_{8_{1}}$ is the rate of its degradation.

Formulas (11) and (12) take into account the fact that in the absence of growth factors at $p_{1_{0}}=0$ and $p_{4_{0}}=0$, the values of *p*_1_ and *p*_4_, respectively, tend to zero and reach maxima at some intermediate ECM density *m* = *m*_2_. In addition, if we assume that the maximum possible cell densities decrease linearly with increasing m and are represented by dependencies: $C_{Smax}=C_{Smax0}\left(1-\frac{m}{m_{\max}}\right)$ and $C_{Cmax}=C_{Cmax0}\left(1-\frac{m}{m_{\max}}\right)$, where $m_{\max}$ is the maximum possible ECM density, then these formulas correspond to a logistic growth model, according to which the cell proliferation rate decreases as their densities approach their maximum values $C_{Smax0}$ and $C_{Cmax0}$. That is, the maximum space available for cell proliferation at any location is modulated by the ECM density at that location.

In addition, if we assume that the maximum possible cell densities decrease linearly with increasing m and are represented by dependencies:

It should also be noted that the proliferation of MSCs and chondrocytes is possible only when the concentration of nutrients n becomes greater than the critical $n_{1}$ which in Equations (1*), (2*) is taken into account by introducing the Heaviside function $H\left(n-n_{1}\right)=\{\begin{array}{c} 0,n\leq n_{1}\\ 1,n>n_{1} \end{array}$. In contrast, at $n_{1}>n$ MSCs and chondrocytes begin to die at rates $p_{3}$ and $p_{5}$, respectively.

The differentiation of MSCs into chondrocytes also does not occur constantly, but only when the condition


(14) $$C_{S}>C_{S_{0}},$$

where $C_{S_{0}}$ is the threshold density of MSCs, determined by the expression


$$C_{S_{0}}=\left(C_{S_{0\max}}-C_{S_{0\min}}\right)e^{-\alpha b}+C_{S_{0\min}},$$

where $C_{S_{0\min}}$ and $C_{S_{0\max}}$ are the minimum and maximum boundaries of the MSCs density; α is the threshold stem cell density reduction factor (66).

Condition (14) in Equations (1*), (2*) is conceded by introducing the Heaviside function


$$H\left(C_{S}-C_{S_{0}}\right)=\{\begin{array}{c} 0,C_{S}\leq C_{S_{0}}\\ 1,C_{S}>C_{S_{0}} \end{array}.$$

Thus, it is not possible to directly take into account changes in the rates of proliferation, differentiation, and death of chondrogenic cells, as well as other significant parameters observed during physical stimulation of the tissue during regenerative rehabilitation, because these changes are characterized by an excessive number of degrees of freedom, due to the fact that they occur under the influence of many interrelated factors. At the same time, the results of our earlier numerical simulation indicate that low-amplitude high-frequency mechanical stimulation promotes the intensification of chondrogenesis and can be used for regenerative rehabilitation of local articular cartilage defects (61). This, as shown above, is also evidenced by the results of numerous *in vitro* studies. The question remains–how does mechanical stimulation *in vivo* contribute to the intensification of chondrogenesis? The answer to this question can be the hypothesis that mechanical stimulation changes not only the micro, but also the macro environment of the cartilage tissue, which contributes to an increase in the proliferation, differentiation, and viability of chondrogenic cells. Therefore, in the first approximation, it can be assumed that in response to mechanical stimulation, the rates of proliferation $p_{1}$, $p_{4}$ and differentiation $p_{2}$ of chondrogenic cells increase to a certain extent, as well as the rates of their death $p_{3}$ and $p_{5}$ decrease, which corresponds to an increase in viability.

Thus, when studying the mathematical model, we will assume that low-amplitude high-frequency mechanical stimulation contributes to a change in the macroenvironment of cartilage tissue. This change occurs continuously and at a certain point in time a certain equilibrium state is reached, described by parameters corresponding to the nature and intensity of stimulation. At the same time, as noted above, *in vitro* cartilage formation increases under the influence of any “correct” physical stimuli that promote proliferation, differentiation of chondrogenic cells, and ECM production. Therefore, it can be assumed that there is a high probability that, as a result of experimental studies, an optimal stimulation method from a practical point of view can be found that contributes to the achievement of the desired macroenvironment of cartilage tissue *in vivo*, which increases the proliferation, differentiation, and viability of chondrogenic cells. Obviously, the parameters of the mathematical model $p_{1},p_{2}$ and $p_{4}$ corresponding to such a macroenvironment will be increased relative to the unstimulated ones, and $p_{3}$ and $p_{5}$ will be reduced, acquiring the values $p_{1}^{*}>p_{1},p_{2}^{*}>p_{2},p_{4}^{*}>p_{4}$ and $p_{3}^{*}<p_{3},p_{5}^{*}<p_{5}$, respectively (14, 43–45). At the same time, the diffusion coefficients of cells, nutrients, growth factors, and ECM elements depend indirectly on the nature of stimulation, since they are dependent on the state variables of the model and change in the process of regenerative rehabilitation along with them.

**Figure 2:**
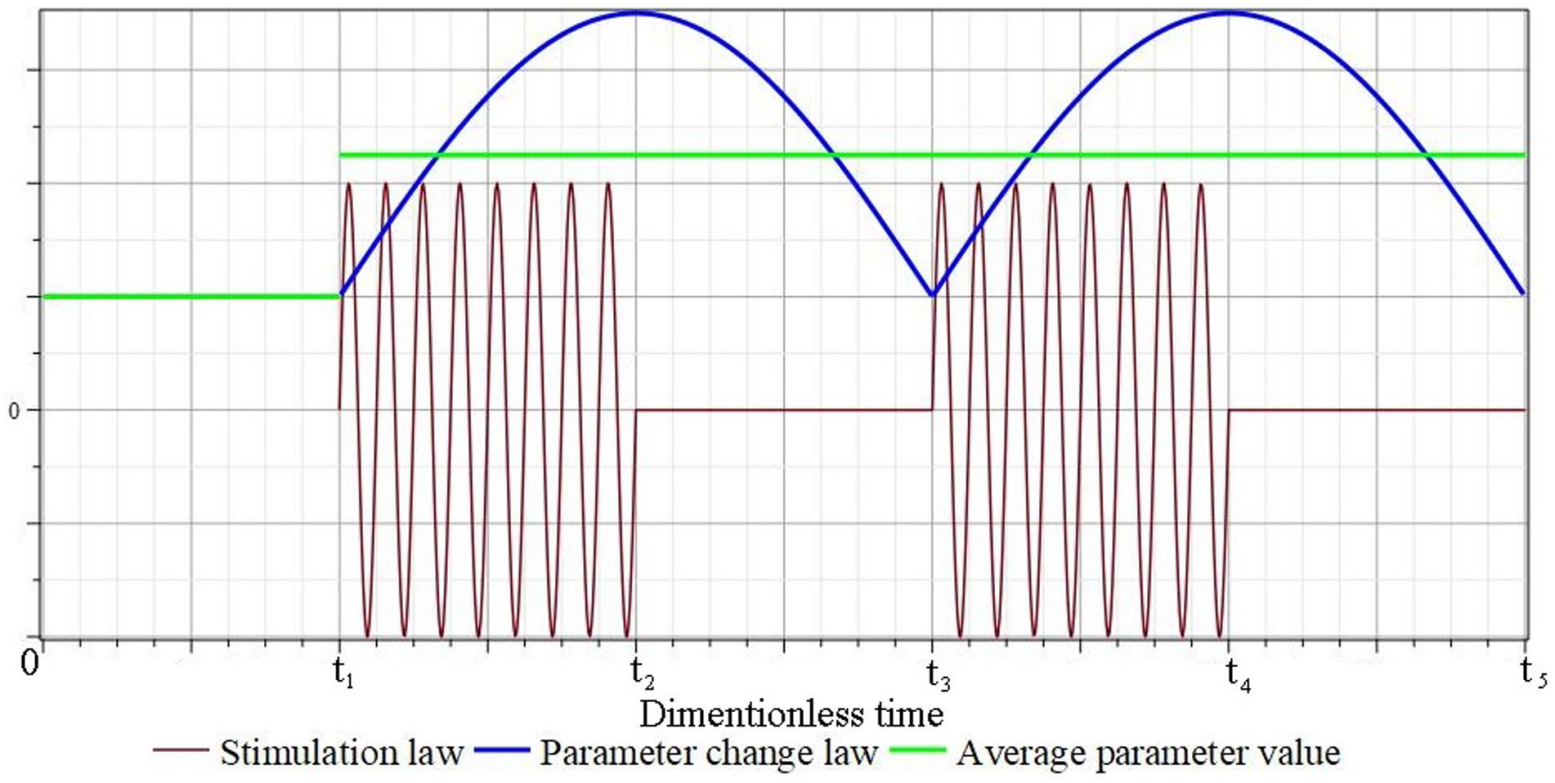
Schematic representation of the mathematical model conditional parameters corresponding to the law of periodic short-term tissue stimulation.

Figure 2 schematically shows graphs of the change in the conditional stimulating effect, the corresponding law of change in the conditional parameter of the mathematical model and its average value. It is assumed that the mechanical stimulation of the tissue in the area of the defect begins after a certain period of time $t_{1}$ and ends at time $t_{2}$. During the time $\Delta t_{12}=t_{2}-t_{1}$ the value of the parameter reaches the maximum/minimum value, and during the time $\Delta t_{23}=t_{3}-t_{2}$ it returns to the minimum/maximum value. In the future, this process is repeated and, to a certain extent, it can be considered close to a process with a certain constant average value of the parameter: $p_{1}^{*}$, $p_{2}^{*}$, etc.

In the future, this process is repeated and, to a certain extent, it can be considered close to a process with a certain constant average value of the parameter: $p_{1}^{*}$, $p_{2}^{*}$, etc.

Numerical experiments by studying the mathematical model of regenerative rehabilitation for local articular cartilage defects (1*, 2*, 3, 4, 5, 6) were performed by the finite element method in the Matlab environment using the built-in m-function “pdepe,” designed to solve systems of parabolic and elliptic partial differential equations with one space variable *x* and time *t*. All solutions were obtained on a 100×100 finite element spatiotemporal grid using the Supercomputer cluster “Afalina” in Sevastopol State University.

The first series of numerical experiments was carried out taking into account the implementation of ASIT/ACIT/ASIT+ACIT with the parameters, the values of which are borrowed in (63, 64) and are given in Appendix 1. In this case, the following were studied:

1. ASIT with boundary Conditions (7), (8) and initial conditions:
  $$\begin{array}{l} C_{S}=0.25\cdot \frac{1-\tanh\left(10^{4}\left(x-0.1\right)\right)}{2},\\ C_{C}=0,n=N_{0},m=m_{3}+m_{s},g=0.01,b=0.01; \end{array}$$
2. ACIT with boundary Conditions (7), (8) and initial conditions:
  $$\begin{array}{l} C_{S}=0,\;C_{C}=0.0001\cdot \frac{1-\tanh\left(10^{4}\left(x-0.1\right)\right)}{2},\\ \;n=N_{0},m=m_{3}+m_{s},g=0.01,b=0.01; \end{array}$$
3. ASIT+ACIT with boundary conditions (7), (8) and initial conditions:
  $$\begin{array}{l} C_{S}=0.25\cdot \frac{1-\tanh\left(10^{4}\left(x-0.1\right)\right)}{2},\\ C_{C}=0.0001\cdot \frac{1-\tanh(10^{4}\left(x-0.1\right)}{2},\\ \;n=N_{0},m=m_{3}+m_{s},g=0.01,b=0.01. \end{array}$$

**Figure 3:**
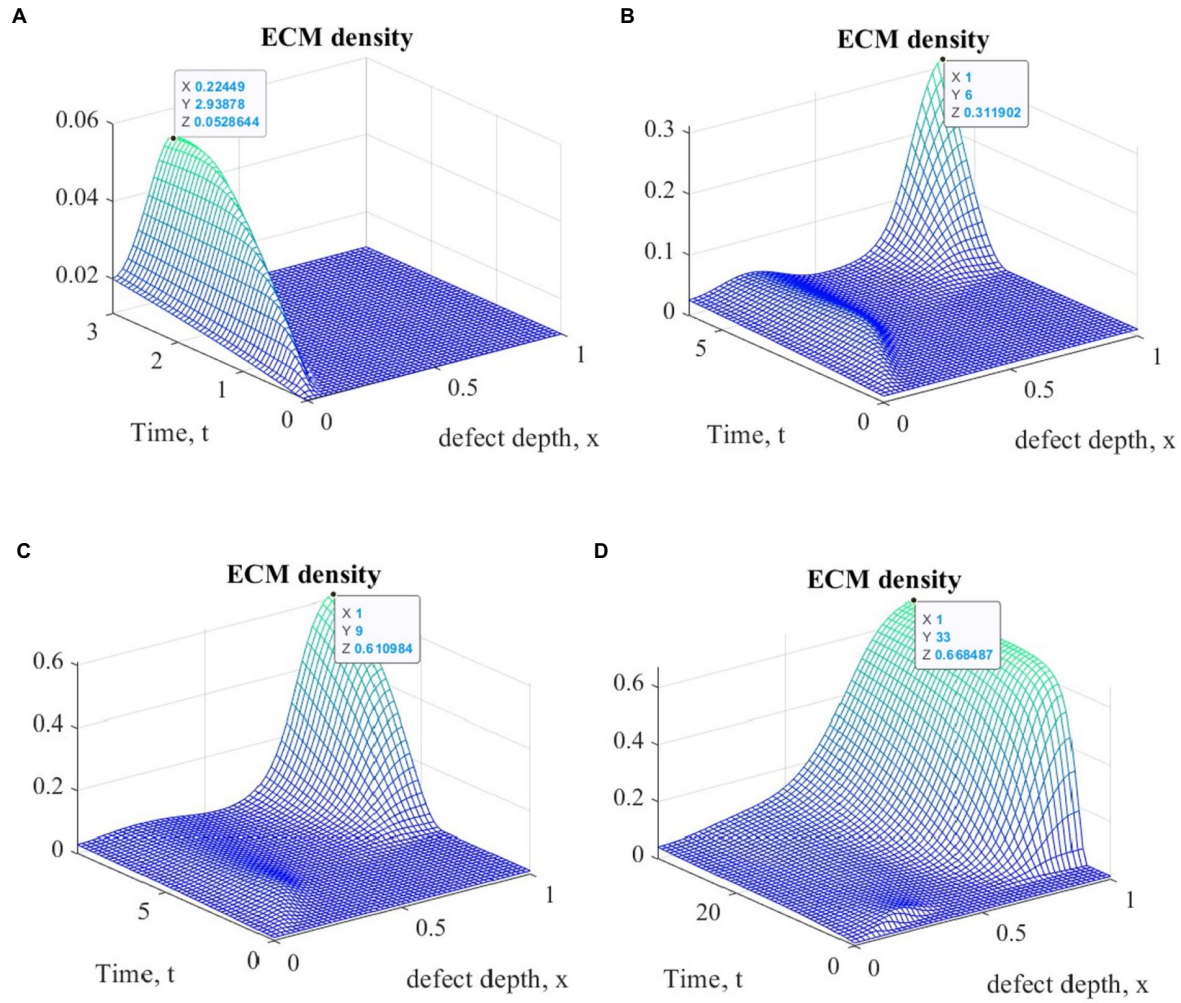
Graphs of ECM density changes at different time points during ASIT implementation: **(A)** *t* = 3 (~1 month); **(B)** *t* = 6 (~ 2 months); **(C)** *t* = 9 (~ 3 months); **(D)** *t* = 33 (~ 12 months).

Figure 3 shows plots of ECM density changes at different time points during ASIT implementation. Similar results were also obtained for other variants of cell therapy.

The general view of the plots of ECM density changes at the same time points is practically the same in the study of all variants of cellular technologies, but their numerical values at the nodes of the finite element grid are different. These differences in the form of maximum ECM density values are shown in Table 1 in dimensionless and real time parameters.

**Table 1:** Maximum values of ECM density at different time points with different options of cell therapy.

Cell therapy option	Maximum ECM density, dimensionless			
	Time elapsed since start of cell therapy			
	Dim.less/Months	Dim.less/Months	Dim.less/Months	Dim.less/Months
	3/1	6/2	9/3	33/12
ASIT	0.0528644	0.311902	0.610984	0.668487
ACIT	0.0571913	0.271928	0.603764	0.668896
ASIT_ACIT	0.0527203	0.312082	0.611128	0.668575

The second series of numerical experiments was performed taking into account the implementation of ASIT/ACIT/ASIT+ACIT with parameters, most of which were also borrowed in (63, 64), show that the proliferation and differentiation of chondrogenic cells under mechanical stimulation increases by (10–30%) (14, 21, 43–45, 67, 68). Two options were considered:


(1) $$p_{10}=15.6,p_{2}=1.3,p_{3}=0.7,p_{40}=p_{400}=0.0156,p_{5}=0.7;$$

(2) $$p_{10}=14.4,p_{2}=1.2,p_{3}=0.8,p_{40}=p_{400}=0.0144,p_{5}=0.8.$$

The assumption we have formulated may not be fully implemented in rehabilitation practice, but it can always be verified as a result of future experimental studies, because the rates of proliferation and differentiation of chondrogenic cells after appropriate rehabilitation procedures can be easily measured in the laboratory. Thus, we create a scientific basis for further research in this direction, which is necessary to confirm the adequacy of the mathematical model we use and improve the technologies for regenerative rehabilitation of articular cartilage defects.

**Figure 4:**
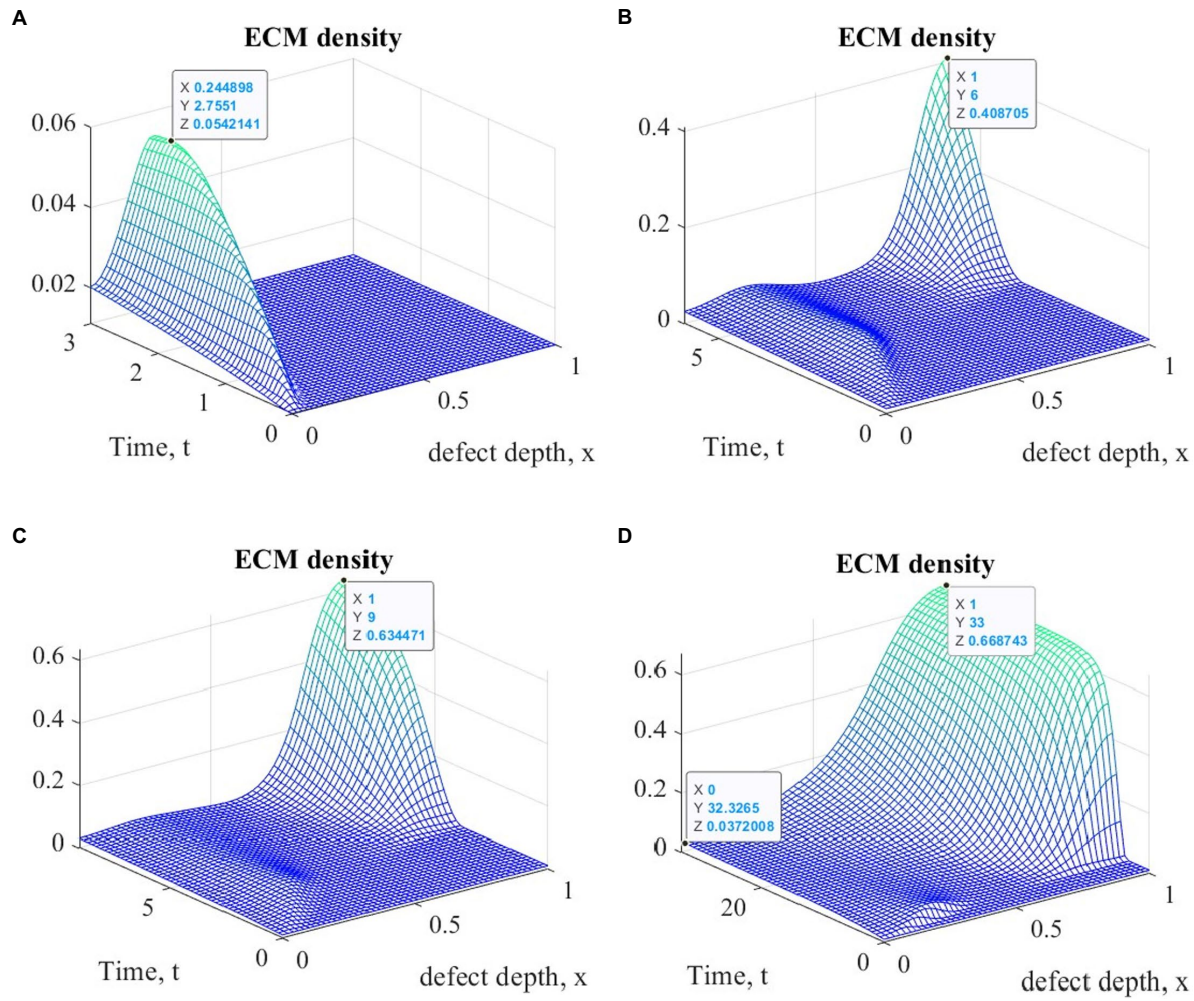
Graphs of changes in ECM density at different time points in the implementation of ASIT with mechanical stimulation with a time delay *t* _1_= 2 (~ 22 days): **(A)** *t* = 3 (~1 month); **(B)** *t* = 6 (~ 2 months); **(C)** *t* = 9 (~ 3 months); **(D)** *t* = 33 (~ 12 months).

Figure 4 shows graphs of changes in ECM density at different time points during the implementation of ACIT with mechanical stimulation with a time delay $t_{1}=2$ (~ 22 days) using the first option of the parameters described above. It is easy to see that they are similar in form to the corresponding graphs obtained in the implementation of ASIT without stimulation. In addition, as in the first series of numerical experiments, their general form at the same time points is almost the same in the study of all options of cellular technologies, with different values at the nodes of the finite element grid. Similar results were also obtained using the second version of the parameters. The maximum values of the densities of the formed ECM at various points in time are given in Table 2 for the two options of the parameters given above.

**Table 2:** Maximum values of ECM density at different time points with different cell therapy options under conditions of tissue mechanical stimulation in the area of the defect.

Cell therapy option	Maximum ECM density, dimensionless			
	Time elapsed since start of cell therapy			
	Dim.less/Months	Dim.less/Months	Dim.less/Months	Dim.less/Months
	3/1	6/2	9/3	33/12
ASIT 1	0.051351	0.436539	0.637694	0.668728
ASIT 2	0.0509713	0.401257	0.630414	0.668703
ACIT 1	0.0542141	0.408795	0.634471	0. 668,743
ACIT 2	0.0549617	0.36955	0.626309	0.668748
ASIT+ACIT 1	0.0515903	0.436741	0.637714	0.668798
ASIT+ACIT 2	0.051433	0.401377	0.630437	0.668706

An elementary analysis of the data presented in Tables 1, 2 allows us to notice that with the parameters of the model adopted on the basis of the cell therapy conditions for articular cartilage with no stimulation, they correspond to the slowest formation of the ECM among other conditions. In this case, the best conditions for the formation of the ECM are achieved with more intense tissue stimulation. At the same time, it should be noted that after a sufficient period of regenerative rehabilitation, the ECM density on the surface of the formed tissue is determined mainly by the restrictions imposed on the values of the model parameters and practically does not depend on the type of cell therapy and the nature of mechanical stimulation.

**Figure 5:**
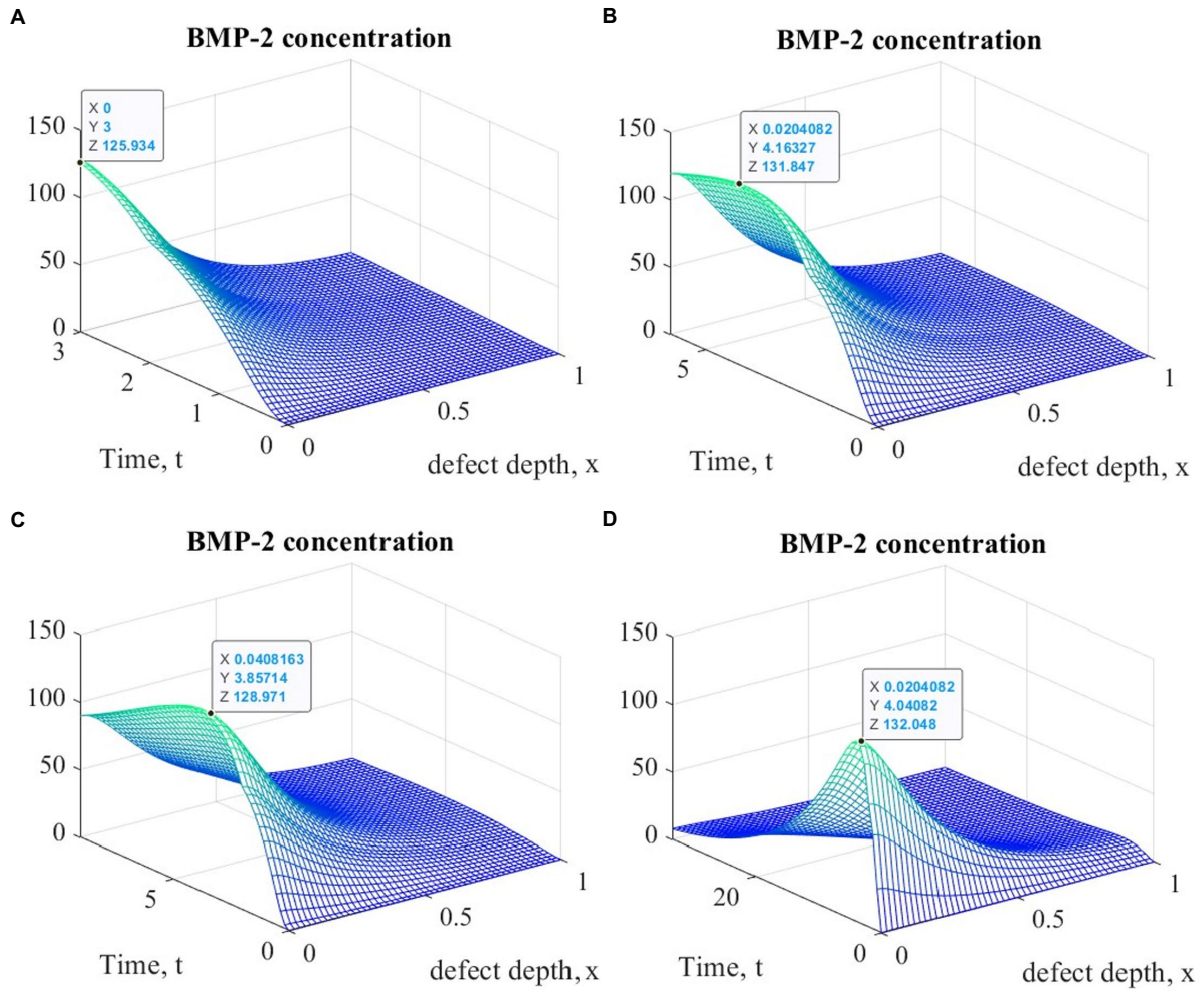
Graphs of changes in BMP-2 concentration at different points in time during the implementation of ACIT with mechanical stimulation with a time delay *t* _1_= 2 (~ 22 days): **(A)** *t* = 3 (~1 month); **(B)** *t* = 6 (~ 2 months); **(C)** *t* = 9 (~ 3 months); **(D)** *t* = 33 (~ 12 months).

**Figure 6:**
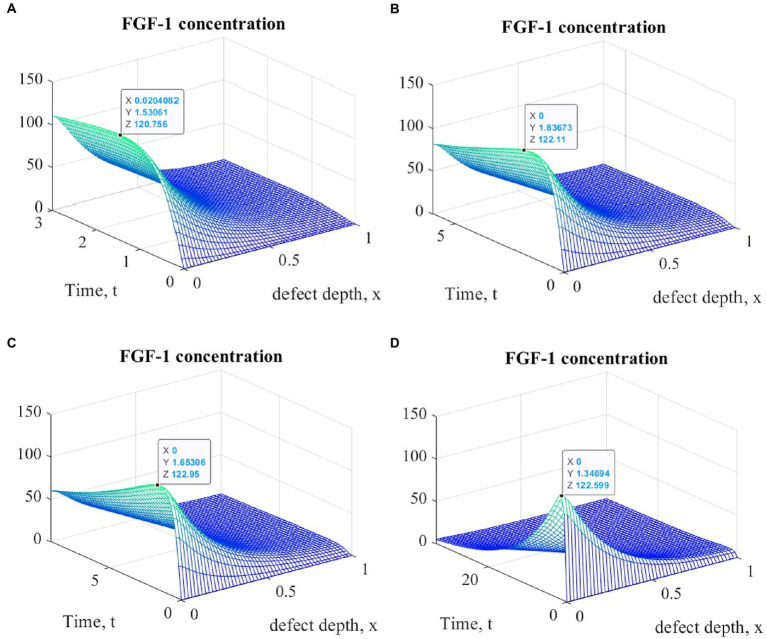
Graphs of changes in FGF-1 concentration at different time points during the implementation of ACIT with mechanical stimulation with a time delay *t* _1_= 2 (~ 22 days): **(A)** *t* = 3 (~1 month); **(B)** *t* = 6 (~ 2 months); **(C)** *t* = 9 (~ 3 months); **(D)** *t* = 33 (~ 12 months).

**Figure 7:**
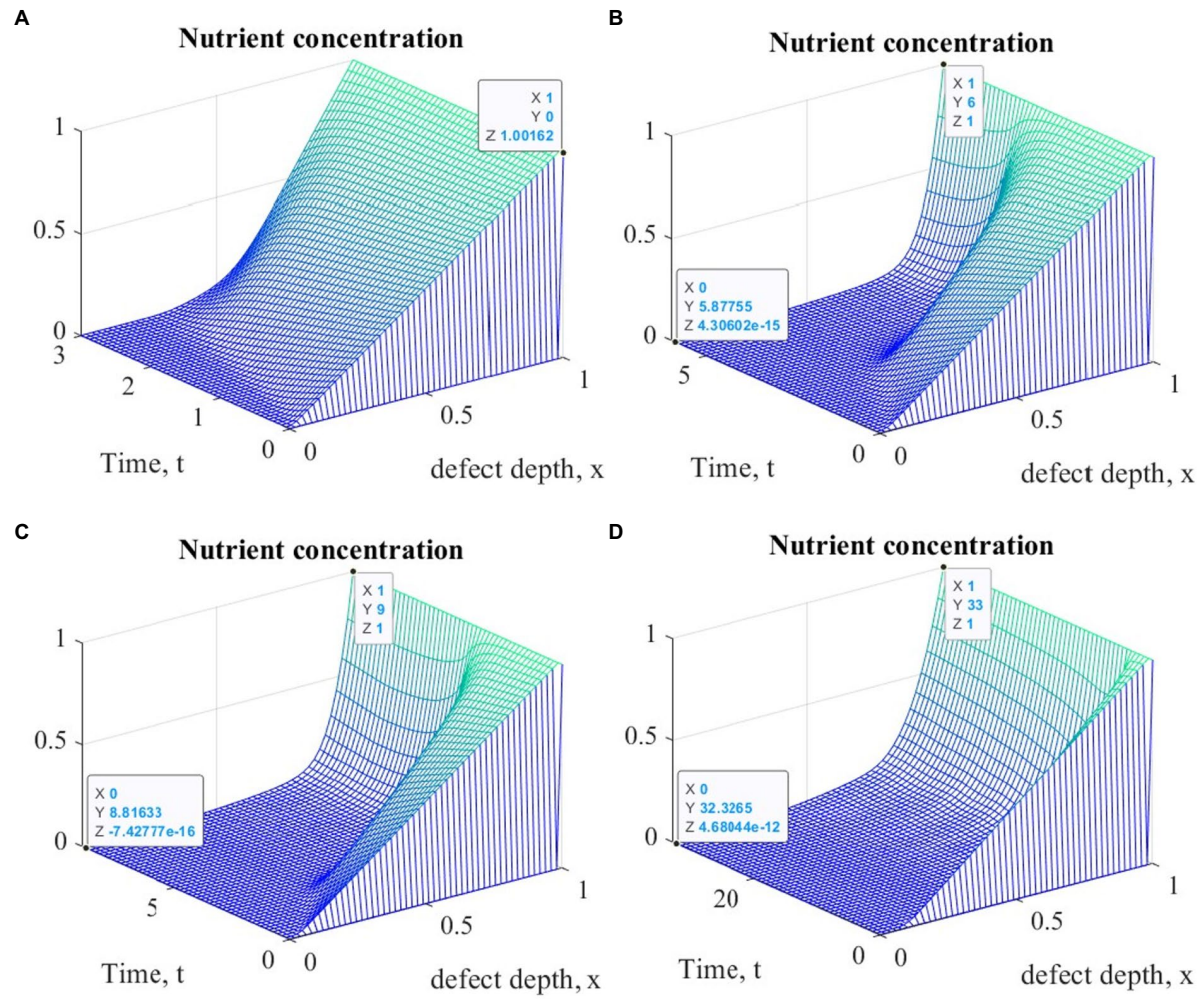
Graphs of changes in nutrient concentration at different points in time during the implementation of ACIT with mechanical stimulation with a time delay *t* _1_= 2 (~ 22 days): **(A)** *t* = 3 (~1 month); **(B)** *t* = 6 (~ 2 months); **(C)** *t* = 9 (~ 3 months); **(D)** *t* = 33 (~ 12 months).

Of particular interest is also the nature of the dynamics of the cartilage tissue regenerative rehabilitation process, which is observed in all types of cell therapy, regardless of the mechanical stimulation intensity. It can be estimated from the results of the other state variables analysis, the graphs of which at various points in time are shown in Figures 5–7.

It is easy to see that the process of matrix formation begins from the side of the subchondral part, while growth factors BMP 2 and FGF 1 play a significant role, the concentrations of which at a certain point in time reach maximum values. These proteins continue to promote the formation of the ECM in the future, but their concentrations significantly decrease and practically tend to zero on the surface of the formed cartilage.

After a certain period of time, more intensive formation of the ECM begins to occur on the surface of the articular cartilage, which is explained by the accumulation in space the necessary amount of nutrients that promote cell proliferation and maintain their viability. Because the mathematical model under study implies a constant replenishment of nutrients from the synovial fluid, this leads to a further increase in the density of the ECM throughout the entire depth of the defect. It can be assumed that in the long term, the ECM will have a gradient structure with a density decreasing towards the subchondral bone.

It should be noted that in this study it was assumed that the maximum diffusion coefficients of all state variables remain constant throughout the entire process of regenerative rehabilitation, since there are no data indicating their change depending on changes in the macroenvironment of the tissue. At the same time, it was assumed that the probabilistic diffusion coefficients change as the ECM density increases. However, given the two-phase structure of the cartilage, it can be assumed that the intensity of diffusion of nutrients under conditions of mechanical stimulation can be significantly increased. Our estimates of this situation show that with an increase in the maximum diffusion coefficient $D_{n}^{*}$, the density of the formed ECM also tends to increase in all types of cell therapy under conditions of mechanical stimulation.

In this work, for the first time, the process of regenerative rehabilitation for cartilage tissue with mechanical stimulation, which provides for some time delay, was studied. The results of the numerical experiments analysis showed that a certain effect associated with this delay is observed and noticeable in the results obtained. However, it should be noted here that delayed rehabilitation procedures for ASIT and ACIT are envisaged in order to achieve the best macroenvironment of the restored tissue, which is not taken into account in the mathematical model used in this study. Therefore, the beginning of mechanical stimulation in a delayed period of time led only to an additive result and practically did not consider the changes that occurred in the tissue during this period of time. Nevertheless, the problem of the mechanical stimulation beginning in the process of cartilage regenerative rehabilitation remains relevant and requires a deeper analysis.

## 4. Conclusion

Modern methods of treating deep articular cartilage lesions are based on the use of various ASIT/ACIT options and are potentially able to provide the formation of new tissue in the area of the defect. However, due to the extremely low regenerative capacity of cartilage due to its morphology, these methods and the technologies underlying them need to be improved. One of the directions that allow eliminating a number of disadvantages inherent in tissue regeneration technologies, including articular cartilage, is called regenerative rehabilitation, which involves the parallel use of regenerative and rehabilitation medicine technologies. Since chondrogenic cells (chondroblasts, young chondrocytes, MSCs) are highly mechanosensitive and proper mechanical stimulation can ensure their differentiation to the phenotype of the main cartilage tissue cells–chondrocytes, it is assumed that this can enhance the regenerative capacity of cartilage tissue and ensure the restoration of its defects. The theories underlying these assumptions are supported by the results of numerous *in vitro* studies. However, it is still not possible to achieve reliable results in the restoration of deep articular cartilage defects *in vivo* using regenerative rehabilitation technologies. One of the reasons is that it is not clear exactly how to stimulate the cartilage tissue in the area of a defect or a tissue-engineered structure populated with chondrogenic cells in order to achieve an adequate chondrogenic response and stimulate the regeneration of new tissue. The answer to this question, or at least the direction in which this answer should be sought, can be obtained as a result of the mathematical models study the regenerative rehabilitation process for articular cartilage. Such models are quite complex, and attempts to take into account all the nuances inherent in tissue regeneration in them can lead to the impossibility of studying them. On the other hand, the results obtained in the study of simplified models may turn out to be far from the true ones. Quite adequate models describing the changes that occur in the tissue during regenerative rehabilitation, the study of which is currently available using finite element methods, are models of the “diffusion–reaction” type. One of the options of such a model was used in this work to study the dynamics of changes in state variables that indirectly characterize the dynamics of the new cartilage tissue formation under conditions of cell therapy and tissue engineering strategies. In particular, we studied the regenerative rehabilitation processes of a local articular cartilage defect using ASIT, ACIT, and ASIT+ACIT both without tissue stimulation and under conditions of delayed mechanical stimulation of varying intensity. The results obtained at the same time indicate that an increase in the proliferation rate of chondrogenic cells seeded in a scaffold placed in the area of the defect leads to a noticeable change in the process of ECM formation. In addition, as a result of numerical experiments, it was found that with an increase in the intensity of mechanical stimulation, accompanied by an increase in the supply of nutrients to the defect area, the process of ECM formation also noticeably intensifies.

The results obtained are of great practical importance, since the rates of proliferation and differentiation of chondrogenic cells after appropriate rehabilitation procedures can be measured in the laboratory. Therefore, such measurements can be used to plan rehabilitation procedures that provide the best tissue repair process.

In further studies, it is planned to study a mathematical model of regenerative rehabilitation with delayed rehabilitation procedures in the short and long term, taking into account all possible options for cellular technologies. Important attention will be paid to the structure and properties of the biodegradable scaffolds used in this case. In addition, in order to obtain modeling results that are of great practical relevance, it is necessary to determine the optimal time delay for the onset of mechanical tissue stimulation in order to ensure its best effect on the process of regenerative rehabilitation.

## Data availability statement

The original contributions presented in the study are included in the article/Supplementary material, further inquiries can be directed to the corresponding authors.

## Author contributions

VLP, AP, and VIP contributed to conception and design of the study. VLP and VIP performed an analysis of literary sources on the research problem. AP performed numerical experiments. All authors contributed to manuscript revision, read, and approved the submitted version.

## Conflict of interest

The authors declare that the research was conducted in the absence of any commercial or financial relationships that could be construed as a potential conflict of interest.

## Publisher’s note

All claims expressed in this article are solely those of the authors and do not necessarily represent those of their affiliated organizations, or those of the publisher, the editors and the reviewers. Any product that may be evaluated in this article, or claim that may be made by its manufacturer, is not guaranteed or endorsed by the publisher.
