# Supplementary material for: In silico evaluation of the mechanical stimulation effect on the regenerative rehabilitation for the articular cartilage local defects
Source: Front Med (Lausanne). 2023 Mar 7;10:1134786. doi: 10.3389/fmed.2023.1134786 (PMC10027915; doi:10.3389/fmed.2023.1134786)
Supplement: Supplementary file 1 [file Table_1.pdf]

Appendix 1. Parameters of the mathematical model

Parameter	Parameter value	Parameter dimension	Dimensionless parameter value
Model State Variables			
C_S – MSC density	$C_S = C_S(x,t)$	$\frac{cells}{mm^3}$	-
C_C – chondrocyte density	$C_C = C_C(x,t)$	$\frac{cells}{mm^3}$	-
m – ECM density	$m = m(x,t)$	$\frac{g}{mm^3}$	-
n – nutrient concentration	$n = n(x,t)$	$\frac{moles}{mm^3}$	-
g – FGF-1 concentration	$g = g(x,t)$	$\frac{g}{mm^3}$	-
b – BMP-2 concentration	$b = b(x,t)$	$\frac{g}{mm^3}$	-
Calculated Model Parameters			
D_S – MSC random motility (diffusion) coefficient	$D_S = D_{S0}\frac{m}{m^2+m_1^2}$	$\frac{mm^2}{hour}$	-
D_C – chondrocyte random motility (diffusion) coefficient	$D_C = D_{C0}\frac{m}{m^2+m_1^2}$	$\frac{mm^2}{hour}$	-
p_1 –MSC proliferation rate	$p_1 = A_m\left(1-\frac{C_S}{C_{Smax}}\right);$	$\frac{cell}{hour}$	-
	$A_m = p_{1_0}\frac{m}{m^2+m_2^2}$; $C_{Smax} = C_{Smax0}\left(1-\frac{m}{m_{max}}\right)$	-	-
p_4 - chondrocyte proliferation rate	$p_4 = B_m\left(1-\frac{C_C}{C_{Cmax}}\right);$	$\frac{1}{hour}$	-
	$B_m = p_{4_0}\frac{m}{m^2+m_2^2} + p_{4_{00}}\frac{g}{g+g_0};$ $C_{Cmax} = C_{Cmax0}\left(1-\frac{m}{m_{max}}\right)$	-	-
C_{S_0} – MSC threshold density	$C_{S_0} = \left(C_{S_{0max}} - C_{S_{0min}}\right)e^{-\alpha b} + C_{S_{0min}}$	$\frac{Nc}{mm^3}$	-
p_8 - ECM synthesis rate	$p_8 = p_{8_0} - p_{8_1}m$	$\frac{g}{mm^3}\cdot\frac{1}{Nc/mm^3}\cdot\frac{1}{hour}$	-
$C_{total,max0}$ – maximum total cell density	$C_{total,max0} = \left(1-\frac{m}{m_{max}}\right)^{-1}(C_{Smax}+C_{Cmax}) = 10^6$	$\frac{Nc}{mm^3}$	
Constant Model Parameters Determining the Biomechanical Environment of the Tissue			
D_{S0} – MSC diffusion constant	$D_{S0} = 2m_1D_S^* = 7.2\times(10^{-9}\div 10^{-8})$	$\frac{mm^2}{hour}\cdot\frac{g}{mm^3}$	0.001 – 0.01
D_S^* – maximum MSC diffusion coefficient	$D_S^* = 3.6\times(10^{-4}\div 10^{-3})$	$\frac{mm^2}{hour}$	-
D_{C0} – chondrocyte diffusion constant	$D_{C0} = 2m_1D_C^*$	$\frac{mm^2}{hour}\cdot\frac{g}{mm^3}$	0.001
D_C^* – maximum chondrocyte diffusion coefficient	$D_C^* = 3.6\times(10^{-4}\div 10^{-3})$	$\frac{mm^2}{hour}$	-
D_n - nutrient diffusion coefficient	$D_n = 4.6$	$\frac{mm^2}{hour}$	100 - 300
D_m – ECM diffusion coefficient	$D_m = 2.5\times10^{-5}$	$\frac{mm^2}{hour}$	0.001 – 0.01
D_g - FGF-1 diffusion coefficient	$D_g = 2\times10^{-3}$	$\frac{mm^2}{hour}$	1.14
D_b - BMP-2 diffusion coefficient	$D_b = 2\times10^{-3}$	$\frac{mm^2}{hour}$	1.14
p_{1_0} – MSC proliferation constant	$p_{1_0} = 2m_2p_1^* = 4\times10^{-6}$	$\frac{g}{mm^3}\cdot\frac{1}{hour}$	12
p_1^* – maximum MSC proliferation rate	$p_1^* = 0.2$	$\frac{cell}{hour}$	-

C_{Smax0} – maximum MSC density	$C_{Smax0} = 0 \div 10^6$	$\frac{Nc}{mm^3}$	0.6
m_1 –intermediate ECM density	$m_1 = 10^{-5}$ (assumed m_{max} /10)	$\frac{g}{mm^3}$	0.1
m_2 –reference ECM density	$m_2 = 10^{-5}$ (assumed m_{max} /10)	$\frac{g}{mm^3}$	0.1
m_{max} - maximum ECM density	$m_{max} = 10^{-4}$	$\frac{g}{mm^3}$	1
p_2 – MSC differentiation rate	$p_2 = 3.75 \times 10^{-3}$	$\frac{1}{hour}$	1
p_3 – MSC death rate	$p_3 = 3.75 \times 10^{-3}$	$\frac{1}{hour}$	1
g_0 - FGF-1 reference concentration	$g_0 = 10^{-10}$	$\frac{g}{mm^3}$	1.0
p_{4_0} – chondrocyte proliferation constant	$p_{4_0} = 2m_2 p_4^* = 4 \times 10^{-9}$	$\frac{g}{mm^3} \cdot \frac{1}{hour}$	0.012
p_4^* - maximum chondrocyte proliferation rate	$p_4^* = 2 \times 10^{-4}$	$\frac{1}{hour}$	-
$p_{4_{00}}$ - chondrocyte proliferation rate (from FGF-1)	$p_{4_{00}} = 2 \times 10^{-4}$	$\frac{1}{hour}$	0.012
C_{Cmax0} - maximum chondrocyte density	$C_{Cmax0} = 0 \div 10^6$	$\frac{Nc}{mm^3}$	0.4
n_1 – critical nutrient concentration	$n_1 = 9.5 \times 10^{-12}$	$\frac{Nm}{mm^3}$	0.1
n_0 – threshold nutrient concentration	$n_0 = 2.3 \times 10^{-11}$	$\frac{Nm}{mm^3}$	0.24 – 0.81
$C_{S_{0max}}$ – maximum threshold MSC density	$\frac{C_{total,max0}}{2}$	$\frac{Nc}{mm^3}$	0.35
$C_{S_{0min}}$ –minimum threshold MSC density	$0.9C_{S_{0max}}$	$\frac{Nc}{mm^3}$	0.315
p_5 – chondrocyte death rate	$p_5 = 3.75 \times 10^{-3}$	$\frac{1}{hour}$	1
p_6 – nutrient uptake constant by MSC	$p_6 = 1.5 \times 10^{-14}$	$\frac{Nm}{Nc \cdot hour}$	10000
p_7 – nutrient uptake constant by chondrocytes	$p_6 = 1.5 \times 10^{-14}$	$\frac{Nm}{Nc \cdot hour}$	10000
p_{8_0} – ECM production constant	$p_{8_0} = 3.75 \times 10^{-13}$	$\frac{g}{mm^3} \cdot \frac{1}{\frac{Nc}{mm^3}} \cdot \frac{1}{hour}$	0.5
$p_{8_{00}}$ - FGF-1 ECM deposition rate	$p_{8_{00}} = 0 \div 1$	-	0.7
p_{8_1} – ECM degradation constant	$p_{8_1} = 3.75 \times 10^{-9}$	$\frac{1}{\frac{Nc}{mm^3}} \cdot \frac{1}{hour}$	1
p_9 - FGF-1 production constant	$p_9 = 10^{-17}$	$\frac{g}{mm^3} \cdot \frac{1}{\frac{Nc}{mm^3}} \cdot \frac{1}{hour}$	26.67
p_{11} - FGF-1 degradation rate	$p_{11} = 5.8 \times 10^{-2}$	$\frac{1}{hour}$	15.4
p_{12} – BMP-2 production constant	$p_{12} = 10^{-17}$	$\frac{g}{mm^3} \cdot \frac{1}{\frac{Nc}{mm^3}} \cdot \frac{1}{hour}$	26.67
p_{13} - BMP-2 degradation rate	$p_{13} = 5.8 \times 10^{-2}$	$\frac{1}{hour}$	15.4
α - threshold stem cell density reduction factor	$\alpha = 10^{10}$	$\frac{1}{g/mm^3}$	100
γ - FGF-1 flux coefficient	$\gamma = 10^{-2}$	$\frac{mm}{hour}$	0.01
χ - BMP-2 flux coefficient	$\chi = 10^{-2}$	$\frac{mm}{hour}$	1
b_0 - BMP-2 reference concentration	$b_0 = 10^{-10}$	$\frac{g}{mm^3}$	-
$C_S^{(0)}$ - initial MSC density	$C_S^{(0)} = 2.5 \times 10^5$(based on 10^6 cells in 20 mm × 20 mm × 10 μm volume)	$\frac{Nc}{mm^3}$	0.25
$C_C^{(0)}$ - initial chondrocytes density	$C_S^{(0)} = 10^2$ (10^{-2}% of total cell density)	$\frac{Nc}{mm^3}$	0.0001

$N_0 = 6.5 \times 10^{-11} \frac{Nm}{mm^3}$ - initial nutrient concentration	$N_0 = (2.85 \div 9.5)10^{-11}$	$\frac{Nm}{mm^3}$	1.0
m_3 – initial ECM density	$m_3 = 10^{-8}$ (assumed m_{max} /10^4)	$\frac{g}{mm^3}$	0.0001
g_{init} − initial FGF-1 concentration	$g_{init} = 10^{-12}$	$\frac{g}{mm^3}$	0.01
b_{init} − initial BMP-2 concentration	$g_{init} = 10^{-12}$	$\frac{g}{mm^3}$	0.01
